# Flexible Endoscopic Laser Surgery for Glottic Carcinoma After Radiotherapy Failure: A First Experience

**DOI:** 10.7759/cureus.106051

**Published:** 2026-03-29

**Authors:** Petru Gurău, Oleg Arnaut, Eusebiu Sencu, Dumitru Sofroni

**Affiliations:** 1 Oncology, Nicolae Testemițanu State University of Medicine and Pharmacy, Chișinău, MDA; 2 Bioinformatics and Computational Medicine Laboratory, Nicolae Testemițanu State University of Medicine and Pharmacy, Chișinău, MDA; 3 National Cancer Registry, Oncological Institute, Chișinău, MDA; 4 Otolaryngology, Nicolae Testemițanu State University of Medicine and Pharmacy, Chișinău, MDA

**Keywords:** endoscopic laser surgery, flexible endoscopy, nd:yag laser, radiotherapy failure, recurrent glottic carcinoma

## Abstract

Background: Surgery is the only salvage treatment for radiotherapy (RT) failures in glottic carcinoma management, and total laryngectomy is performed in the majority of cases, resulting in a substantial decline in the quality of life for patients. Transoral CO2 laser microsurgery (TOLMS), as a salvage treatment for RT failures, demonstrated its efficiency, albeit more inferior compared to treatment results obtained in primary glottic carcinoma, but has some limitations, predominantly associated with difficult anatomy and inadequate glottis exposure and also because of mandatory larynx suspension and general anesthesia with myorelaxation that present risks or contraindications for some categories of patients. Flexible endoscopic laser surgery (FELS) can overcome some of the limitations of TOLMS, being a minimally invasive therapeutic option for this category of patients. The study's purpose was to evaluate the long-term (five-year) effectiveness of FELS in treating recurrent/persistent glottic carcinoma after RT failure.

Methods: FELS was performed in nine patients with recurrent (four) and persistent (five) early-stage (T1a: two; T1b: one; T2: six) glottic carcinoma after RT failure (males: eight; females: one), aged 47-66 (mean: 55.4±7.8). FELS under local anesthesia with spontaneous ventilation was performed in five patients (55.6%), and the rest of the patients were operated on under general anesthesia with superimposed high-frequency jet ventilation (SHFJV). Tumor ablation by neodymium-doped yttrium aluminum garnet (Nd:YAG) laser was performed in all the cases, and adjuvant RT (20-22 Gy) was performed in two cases of persistent T2 tumors.

Results: Five-year overall survival and ultimate disease control, including salvage treatment, were obtained in six patients (66.7%), cure with larynx preservation was obtained in five patients (55.6%), disease-free survival was obtained in five cases (55.6%), and ultimate local control with FELS alone was obtained in five cases (55.6%). All three patients with the T1 stage of disease were alive, free of disease, with the preserved larynx due to FELS alone.

Conclusion: FELS can be considered an efficient method of treating recurrent and persistent T1-T2 glottic carcinoma after RT failure, offering a minimally invasive surgical alternative for cure with larynx preservation, especially for patients with risks/contraindications to general anesthesia and transoral microsurgery.

## Introduction

Larynx carcinoma represents about 30-50% of all head and neck malignancies, with 75% of the tumors being located in the glottic portion of the larynx [1-3]. At present, external beam radiotherapy (RT) is still largely used in many institutions as the first-line treatment for early glottic carcinoma, demonstrating good oncological results. The tumor recurrence rate after RT varies between 5% and 20% for T1 lesions and between 25% and 50% for T2 lesions [4,5]. The impossibility of repeating RT for a recurrent tumor is a major disadvantage of this treatment method. Consequently, surgery is the only salvage treatment for RT failures, and total laryngectomy is performed in the majority (75%) of cases [6], resulting in a substantial decline in the quality of life of the patients. 

Transoral CO2 laser microsurgery (TOLMS), being applied in recent decades as a salvage treatment for RT failures in early glottic carcinoma, has demonstrated its efficiency, albeit more inferior compared to treatment results obtained in primary glottic carcinoma [7], but has some limitations, predominantly associated with difficult anatomy and inadequate exposure of the glottis and also because of mandatory larynx suspension and general anesthesia with myorelaxation that present risks or contraindications for some categories of patients (e.g., ischemic cardiovascular disease, dental mobility or prosthetic work in the anterior maxillary region) [7-13].

Flexible endoscopic laser surgery (FELS) can overcome some of the abovementioned limitations of TOLMS, being a minimally invasive therapeutic option for this category of patients. The objective of this study was to assess the long-term (five-year) effectiveness of FELS in treating glottic carcinoma after RT failure.

## Materials and methods

The retrospective study was conducted at Timofei Moșneaga Republican Clinical Hospital in Chișinău, Republic of Moldova, after obtaining approval from the Institutional Ethics Committee of Nicolae Testemițanu State University of Medicine and Pharmacy (approval number: 6). FELS was performed in nine patients with recurrent/persistent glottic carcinoma after RT failure. All surgical interventions were performed by a single surgeon. Written informed consent was obtained from the patients. Consecutive patients with recurrent/persistent T1-T2 N0 M0 glottic carcinoma after RT failure who could be followed up for five years after the endoscopic intervention were included in the study.

**Figure 1:**
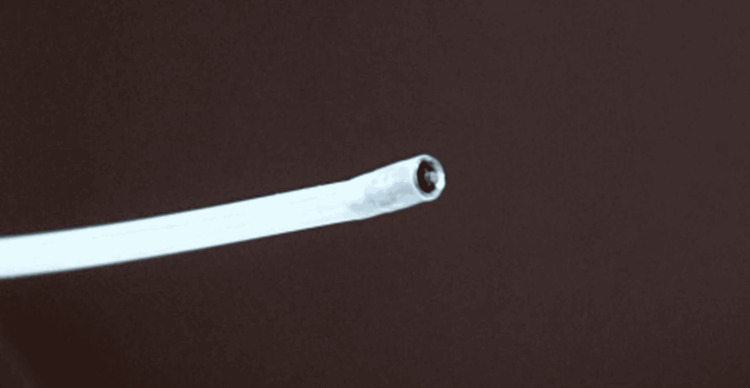
Flexible Nd:YAG laser guide Nd:YAG: neodymium-doped yttrium aluminum garnet

FELS was performed using a therapeutic bronchoscope (2.6-3 mm working channel) and flexible guide-based neodymium-doped yttrium aluminum garnet (Nd:YAG) laser (model: LTN-102 (Russia); wavelength: 1064 nm) (Figure 1).

**Figure 2:**
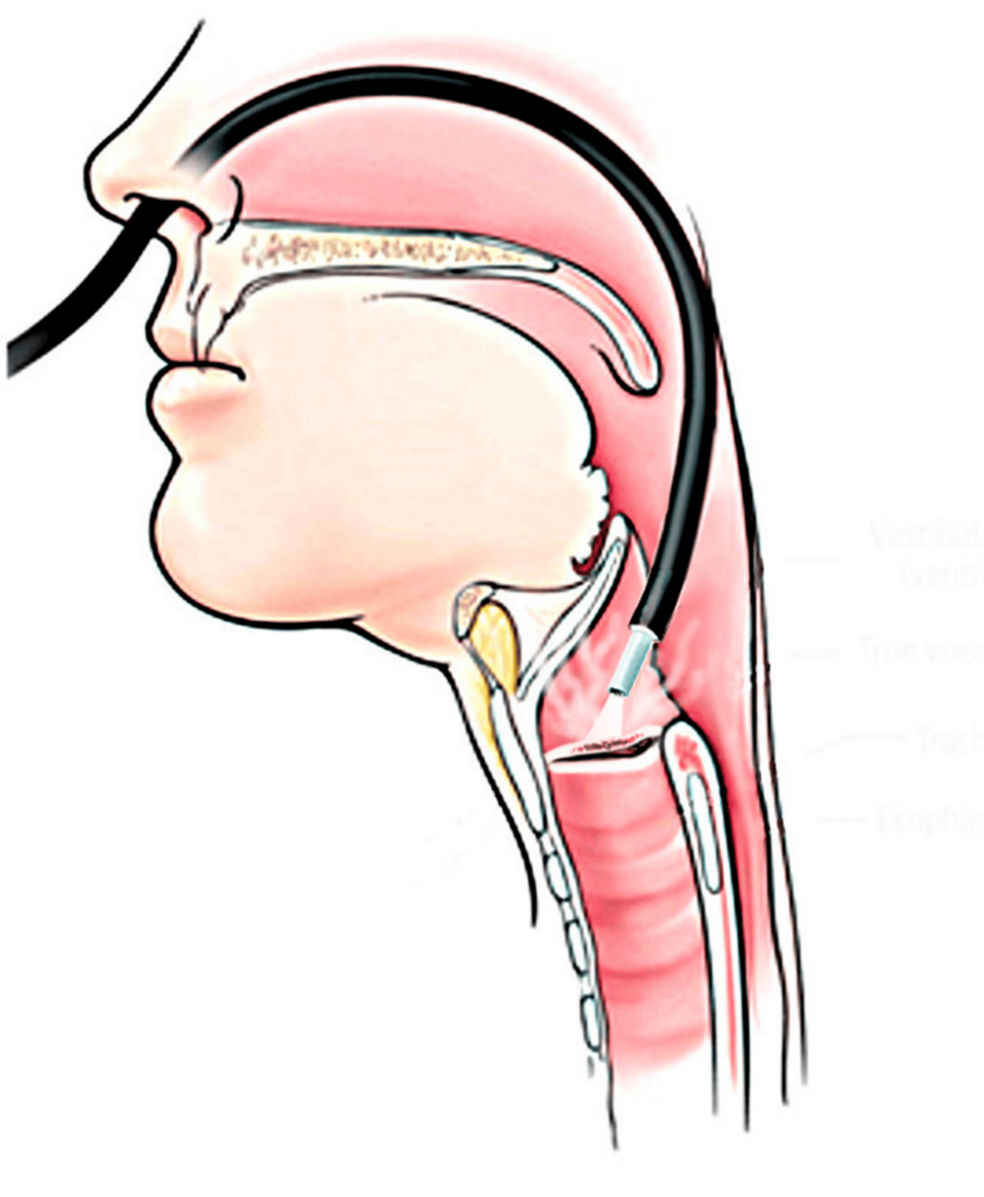
Transnasal approach to the glottic lesion The figure was created using Adobe Photoshop without AI assistance (San Jose, California, United States).

When the intervention was performed with spontaneous ventilation under local anesthesia and moderate intravenous sedation, patients were treated in a sitting position. A flexible bronchoscope was introduced into the larynx transnasally after local anesthesia of the nasal and pharyngeal mucosa with 10% lidocaine spray. Topical anesthesia of the larynx mucosa was performed with lidocaine 2% instilled through a catheter introduced in the working channel of the bronchoscope during phonation, and a flexible laser guide was delivered to the lesion through the working channel of the bronchoscope (Figure 2).

**Figure 3:**
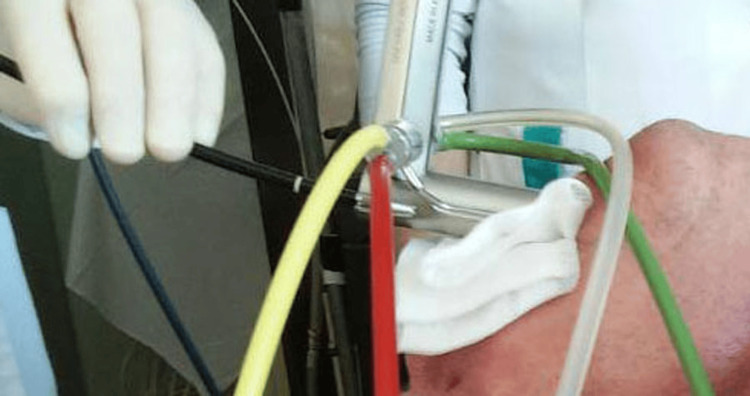
Flexible bronchoscope, together with a flexible laser guide, is introduced through the rigid suspensive jet laryngoscope

When the intervention was performed under general anesthesia with superimposed high-frequency jet ventilation (SHFJV), a flexible bronchoscope, together with a flexible laser guide, was introduced through the rigid suspensive jet laryngoscope ("Carl Reiner", Austria) (Figure 3).

**Figure 4:**
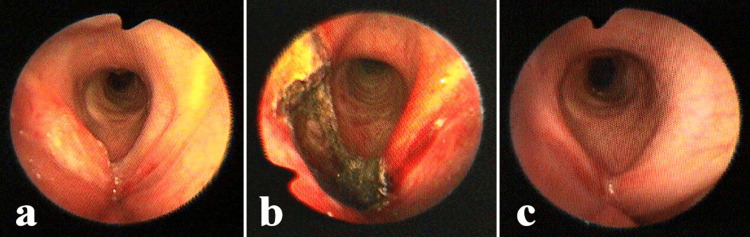
Recurrent carcinoma of the right vocal fold (a) Before the treatment. (b) Immediately after Nd:YAG laser ablation. (c) One year after endoscopic surgery. Nd:YAG: neodymium-doped yttrium aluminum garnet

Photoablation of tumors was performed in continuous near non-contact mode with power settings of 20-40W (Figure 4). Awake procedures with spontaneous ventilation were chosen in cases of contraindications or major risk for general anesthesia with myorelaxation and/or transoral microsurgery and the patient's desire to undergo an awake endoscopic surgery.

The following criteria were analyzed for the evaluation of the long-term (five-year) oncologic efficacy of FELS: ultimate disease control (UDC) (free of primary tumor, regional, and distant metastases), overall survival rate (OS) (alive five years post-surgery), disease-free survival (DFS) (alive five years post-surgery without local or regional recurrence), ultimate disease control with FELS alone (UDC FELS) (free of primary and metastatic tumor after FELS only), cure with larynx preservation (CLP) (free of disease with preserved larynx), death of disease (DD) (death from primary or metastatic tumor), and local recurrence (LRc): LRc included recurrent primary tumors (revealed over six months after the treatment) and persistent primary tumors (revealed within six months after the treatment).

## Results

Nine patients with recurrent/persistent T1-T2 glottic carcinoma after RT failure were included in the study: eight males and one female aged 47-66 years (mean: 55.4±7.8 years). The histological structure of the tumors was represented by squamous cell carcinoma in all the cases. According to the tumor extent, two patients had T1a stage, one patient had T1b stage, and six patients had T2 stage of the disease (due to supraglottic and/or subglottic extension of the tumor). The anterior commissure involvement was registered in six cases (66.7%).

In seven cases, only FELS was performed, and in two patients (T2 persistent carcinoma), postoperative RT in a dosage of 20-22 Gy was also offered. The reasons for applying adjuvant RT for those two cases of persistent tumors were completing the preoperative dosage of RT (40 and 46 Gy, respectively) and consolidation of the therapeutic effect of FELS. 

In eight observations, treatment was realized in one session, and for one patient, in two sessions. Awake interventions with spontaneous ventilation were performed in five patients (55.6%), and general anesthesia with SHFJV was used in four cases.

No complications during or after endoscopic interventions were registered. Five-year overall survival and ultimate disease control, including salvage treatment, were obtained in six patients (66.7%). Disease-free survival was achieved in five cases (55.6%). Primary tumor recurrence was registered in three cases (33.3%). Successful salvage treatment (total laryngectomy) for tumor recurrence was registered in one patient. Death of primary tumor progression was registered in two cases (22.2%) after 48 and 53 months from endoscopic intervention. In one case, after seven months from FELS, the patient died of a brain tumor that was considered metastatic, though there was no firm certainty about the association between laryngeal and brain tumors. Cure with larynx preservation was obtained in five cases (55.6%). Ultimate disease control with FELS alone was achieved in five patients (55.6%). All three patients with T1 disease were alive, cured of the disease with preserved larynx due to FELS alone (Table 1). 

**Table 1:** Five-year results of FELS for recurrent/persistent glottic carcinoma after RT failure related to tumor stage n: number of patients; UDC: ultimate disease control; OS: overall survival rate; DFS: disease-free survival; UDC FELS: ultimate disease control with FELS alone; CLP: cure with larynx preservation; DD: death of disease; LRc: local recurrence; FELS: flexible endoscopic laser surgery; RT: radiotherapy

Results	Overall (n=9)	T1a (n=2)	T1b (n=1)	T2 (n=6)
UDC/OS	6/9 (66.7%)	2/2 (100%)	1/1 (100%)	3/6 (50%)
DFS	5/9 (55.6%)	2/2 (100%)	1/1 (100%)	2/6 (33.3%)
UDC FELS	5/9 (55.6%)	2/2 (100%)	1/1 (100%)	2/6 (33.3%)
CLP	5/9 (55.6%)	2/2 (100%)	1/1 (100%)	2/6 (33.3%)
DD	3/9 (33.3%)	0/2 (0%)	0/1 (0%)	3/6 (50%)
LRc	3/9 (33.3%)	0/2 (0%)	0/1 (0%)	3/6 (50%)

## Discussion

RT is still the preferred method of treating early glottic carcinoma in many institutions. The tumor recurrence rate after RT varies between 5% and 20% for T1 lesions and between 25% and 50% for T2 lesions [4,5]. The disadvantages of RT include radioresistance of some tumors, reduced efficiency in bulky lesions, verrucous carcinoma, anterior commissure affection, and the impossibility of reusing RT for a recurrent tumor [7,14,15].

Therefore, surgery is the only treatment for RT failures. Though open partial laryngectomy (OPL) can be used for treating RT failures, the method has disadvantages that include major surgical trauma caused by the cutting of normal anatomical structures (muscles, nerves, cartilage, vessels), resulting in pain and postoperative edema, temporary tracheostomy (5-18 days), temporary nasogastric tube placement because of deglutition impairment and aspiration episodes, long hospitalization period (22-35 days), and high rate (up to 51%) of postoperative complications [12,16-18]. The incision of thyroid cartilage that was previously irradiated favors the apparition of severe postoperative complications, such as chondritis, chondronecrosis, and fistula [19]. Consequently, in case of RT failure, total laryngectomy is performed in the majority (75%) of cases [6], resulting in a substantial decline in the quality of life of the patients. TOLMS with CO2 laser is considered the gold standard in the surgical management of early glottic carcinoma that replaced OPL as a primary treatment modality, having such advantages as minor surgical trauma, preserving the integrity of cartilaginous skeleton of the larynx and avoiding tracheostomy, short treatment duration, the possibility to be repeated, and preserving of all salvage treatment options in case of tumor recurrence [7,9,10,18,20].

TOLMS is not always applicable because of some limitations that are predominantly associated with difficult anatomy and inadequate tumor exposure and also because of mandatory larynx suspension and general anesthesia with myorelaxation that present risks or contraindications for some categories of patients (e.g., ischemic cardiovascular disease, dental mobility, or prosthetic work in the anterior maxillary region) [7-13].

In contrast to primary glottic carcinoma, the experience of using TOLMS for treating recurrent glottic carcinoma after RT failure is still limited. Inferior results of TOLMS in treating recurrent glottic carcinoma compared to primary carcinoma, a higher treatment complication rate, and frequent necessity of repeated procedures for disease control have been reported [7,21]. Blakeslee et al., analyzing a series of 15 patients, reported local control of 40% in treating recurrent T1 glottic carcinoma after RT failure [22]. Ramakrishnan et al., in a review and meta-analysis publication based on 11 studies, mentioned 56.9% local control after the first intervention [23]. Weiss et al., in a retrospective study, reported a five-year local control of 57.5% after performing TOLMS in 93 patients with recurrent early glottic carcinoma [24]. Roedel et al., analyzing the oncological outcomes of TOLMS for recurrent early glottic carcinoma after primary RT, reported a 42% cure rate after the first intervention [19]. Russo et al., in a recent review and meta-analysis publication, analyzing the results of salvage TOLMS after primary RT for 235 patients, reported a summarized five-year local control of 39.1% [25].

The limitations of TOLMS can be overcome by FELS. In the available English literature, we have not found any studies about using FELS for treating recurrent glottic carcinoma after primary RT failure. To our knowledge, this is the first series of patients treated by FELS and followed up for five years. The laser ablation technique's disadvantage is the impossibility of assessing the margins of the resected specimen. However, close follow-up and the "wait-and-see" strategy for the early detection of possible tumor recurrence can partially compensate for this. We recommend postoperative follow-up flexible laryngoscopy exams once a month during the first year after surgery, once in two months during the second year, once in three months during the third year, once in six months during the fourth and fifth years, and once a year after five years. There is no substantial evidence behind adjuvant reirradiation after salvage surgery. The rationale for such a decision in two of our cases was the following: the mentioned two patients did not receive a complete dosage of RT before salvage endoscopic treatment, so a persistent tumor was detected after the first half of the RT course. Thus, completing the RT program (up to 60-70 Gy) after substantial surgical cytoreduction was considered rational for increasing the chances of local control. The limitations of the study, which include its retrospective nature, the small number of observations, and the absence of a control group, do not permit categorical affirmations. Nevertheless, obtaining disease-free survival, cure with larynx preservation, and ultimate disease control by FELS alone at 55.6% allows us to state that the oncological efficiency of FELS is comparable to that of TOLMS and the method deserves to be applied as a salvage treatment after RT failure, offering patients a chance to preserve their larynx.

## Conclusions

From the oncological perspective, FELS can be considered an efficient method for treating recurrent/persistent T1-T2 glottic carcinoma. The method deserves to be used as a minimally invasive surgical alternative for disease control with larynx preservation, primarily for patients with risks/contraindications to general anesthesia with myorelaxation and TOLMS. Prospective studies involving a larger number of patients are needed to validate the proposed approach and provide categorical statements. 
